# Expression of *adhA* from different organisms in *Clostridium thermocellum*

**DOI:** 10.1186/s13068-017-0940-8

**Published:** 2017-11-30

**Authors:** Tianyong Zheng, Jingxuan Cui, Hye Ri Bae, Lee R. Lynd, Daniel G. Olson

**Affiliations:** 1 Department of Biological Sciences, Dartmouth College, Hanover, NH 03755 USA; 2 Bioenergy Science Center, Oak Ridge National Laboratory, Oak Ridge, TN 37830 USA; 3 Thayer School of Engineering, Dartmouth College, 14 Engineering Drive, Hanover, NH 03755 USA

**Keywords:** Consolidating bioprocessing, *Clostridium thermocellum*, Alcohol dehydrogenase, *adhA*, Biofuel, Ethanol

## Abstract

**Background:**

*Clostridium thermocellum* is a cellulolytic anaerobic thermophile that is a promising candidate for consolidated bioprocessing of lignocellulosic biomass into biofuels such as ethanol. It was previously shown that expressing *Thermoanaerobacterium saccharolyticum adhA* in *C. thermocellum* increases ethanol yield.In this study, we investigated expression of *adhA* genes from different organisms in *Clostridium thermocellum*.

**Methods:**

Based on sequence identity to *T. saccharolyticum adhA*, we chose *adhA* genes from 10 other organisms: *Clostridium botulinum*, *Methanocaldococcus bathoardescens*, *Thermoanaerobacterium ethanolicus*, *Thermoanaerobacter mathranii*, *Thermococcus* strain AN1, *Thermoanaerobacterium thermosaccharolyticum*, *Caldicellulosiruptor saccharolyticus*, *Fervidobacterium nodosum*, *Marinitoga piezophila*, and *Thermotoga petrophila*. All 11 *adhA* genes (including *T. saccharolyticum adhA*) were expressed in *C. thermocellum* and fermentation end products were analyzed.

**Results:**

All 11 *adhA* genes increased *C. thermocellum* ethanol yield compared to the empty-vector control. *C. botulinum* and *T. ethanolicus adhA* genes generated significantly higher ethanol yield than *T. saccharolyticum adhA*.

**Conclusion:**

Our results indicated that expressing *adhA* is an effective method of increasing ethanol yield in wild-type *C. thermocellum*, and that this appears to be a general property of *adhA* genes.

## Background

*Clostridium thermocellum* is a cellulolytic anaerobic thermophile that is considered to be a promising candidate for consolidated bioprocessing of lignocellulosic biomass, into biofuels such as ethanol, due to its native ability to solubilize lignocellulose [1]. A key limitation of this organism is that it produces ethanol only at low yield (20% of the theoretical maximum) [2]. Strategies to increase ethanol yield in *C. thermocellum* include deleting the pathways for acetic acid, lactic acid, and hydrogen production [3–6], and introducing heterologous genes from ethanol production pathways in other organisms [2, 7], such as *Thermoanaerobacterium saccharolyticum.* Recently, it was shown that AdhA plays an important role in ethanol production in strains of *T. saccharolyticum* engineered for homoethanol production [8]. This enzyme was subsequently expressed in *C. thermocellum* and shown to increase ethanol yield and titer by 40% [3]. In this study, we chose *adhA* genes from 10 additional organisms, expressed them in *C. thermocellum* and observed the effect on ethanol production.

## Methods

### Plasmid and strain construction

Plasmids used for *adhA* expression in *C. thermocellum* are listed in Table 1. Plasmids were constructed based on the *C. thermocellum* expression plasmid pDGO144 as previously described [9]. The Clo1313_2638 promoter [9] and *adhA* gene were cloned into the *Hin*dIII site of pDGO144 using standard molecular biology techniques. The correct reading frame and sequence of each *adhA* gene in the resulting plasmids in Table 1 were confirmed by Sanger Sequencing (GENEWIZ). Complex medium CTFÜD [10] was used to culture wild-type *C. thermocellum.* Plasmids expressing *adhA* genes were transformed into wild-type *C. thermocellum* using the transformation protocol as previously described [10]. Selection was carried out using thiamphenicol at a final concentration of 6 µg/ml. Single colonies were picked and re-inoculated into CTFÜD medium containing 6 µg/ml thiamphenicol; cultures were saved for further analysis. The presence of *adhA* genes in the cultures was confirmed by PCR. Primers used for the confirmation are Fwd: GACGAAAAAGCCGATGAAG, Rev: CCTTTTTTAAAAGTCAATCCCG. The size of the PCR product was used to confirm *adhA* insertion: the PCR product of the empty vector is 178 bp, and the PCR product containing the *adhA* gene insertion is ~ 1400 bp (with slight variation due to differences in lengths of the *adhA* genes).

**Table 1:** Strains and plasmids used in this work

Strain ID	Plasmid ID	Source of *adhA* on the plasmid	Source organism abbreviation	Sequence identity to *Tsac* AdhA	Source organism optimal growth temperature (°C)	GenBank accession number
LL1525	pCBcth1	*Thermoanaerobacter mathranii*	*Tmat*	86%	70–75 [17]	MG026506
LL1526	pCBcth2	*Thermoanaerobacterium ethanolicus*	*Teth*	88%	70 [18]	MG026510
LL1527	pCBcth3	*Clostridium botulinum*	*Cbot*	62%	37 [19]	MG026514
LL1528	pCBcth4	*Thermococcus strain AN1*	*Ther*	65%	75–80 [20]	MG026513
LL1529	pCBcth7	*Thermotoga petrophila*	*Tpet*	60%	80 [21]	MG026511
LL1530	pCBcth8	*Methanocaldococcus bathoardescens*	*Mbat*	57%	82 [22]	MG026508
LL1531	pCBcth9	*Marinitoga piezophila*	*Mpie*	61%	65 [23]	MG026515
LL1532	pCBcth12	*Thermoanaerobacterium thermosaccharolyticum*	*Tthe*	90%	68 [24]	MG026507
LL1533	pCBcth13	*Fervidobacterium nodosum*	*Fnod*	67%	65–70 [25]	MG026509
LL1534	pCBcth14	*Caldicellulosiruptor saccharolyticus*	*Csac*	76%	70 [26]	MG026512
LL1535	pDGO144 [9]^a^	NA	NA	NA	55	
LL1536	pCBcth17	*Thermoanaerobacterium saccharolyticum*	*Tsac*	100%	60 [27]	MG026516

*NA* not applicable

^a^The empty vector pDGO144 is also known as pCBcth15

### Fermentations and end-product analysis

For fermentation end-product analysis, strains were transferred three times in defined MTC-5 medium [11] with 4.7 g/l cellobiose at 1% inoculum (v/v). End-product measurements were taken on the 3rd transfer. Cultures were grown in Corning™ Falcon™ 15 ml Conical Centrifuge Tubes and incubated anaerobically without shaking at 55 °C for 72 h. Upon harvesting, cultures were prepared as previously described for HPLC (High-Pressure Liquid Chromatography) analysis [8]. Ethanol yield was calculated as the percentage of theoretical yield based on the amount of ethanol produced and substrate consumed: $$\left[ {{\text{Yield ethanol }}\left( \%{{\text{ maximum theroretical}}} \right) = \frac{{\rm Amount of ethanol produced (mM)}}{4*{\rm Amount of cellobiose consumed (mM)}}} \right]$$. Carbon balance was calculated based on the fermentation products measured as previously described [12]: $$\left[ {{\text{Carbon balance }}\left( {\text{\% }} \right) = \frac{{\left[ {\rm Acetate} \right] + \left[ {\rm Ethanol} \right] + \left[ {\rm Lactate} \right]\left( {\rm mM} \right)}}{{4*\left[ {\rm cellobiose consumed} \right]\left( {\rm mM} \right)}}} \right].$$

### Phylogenetic analysis

The amino acid sequences of different AdhA proteins were aligned using CLC Main Workbench 7.7.3, and a phylogenetic tree was created using the Neighbor Joining algorithm. Distance is expressed as substitutions per 100 amino acids; multiple substitutions at the same site were corrected for using the Kimura method. Bootstrap analysis was performed with 1000 replicates.

## Results and discussion

### *adhA* genes from different organisms

**Fig. 1:**
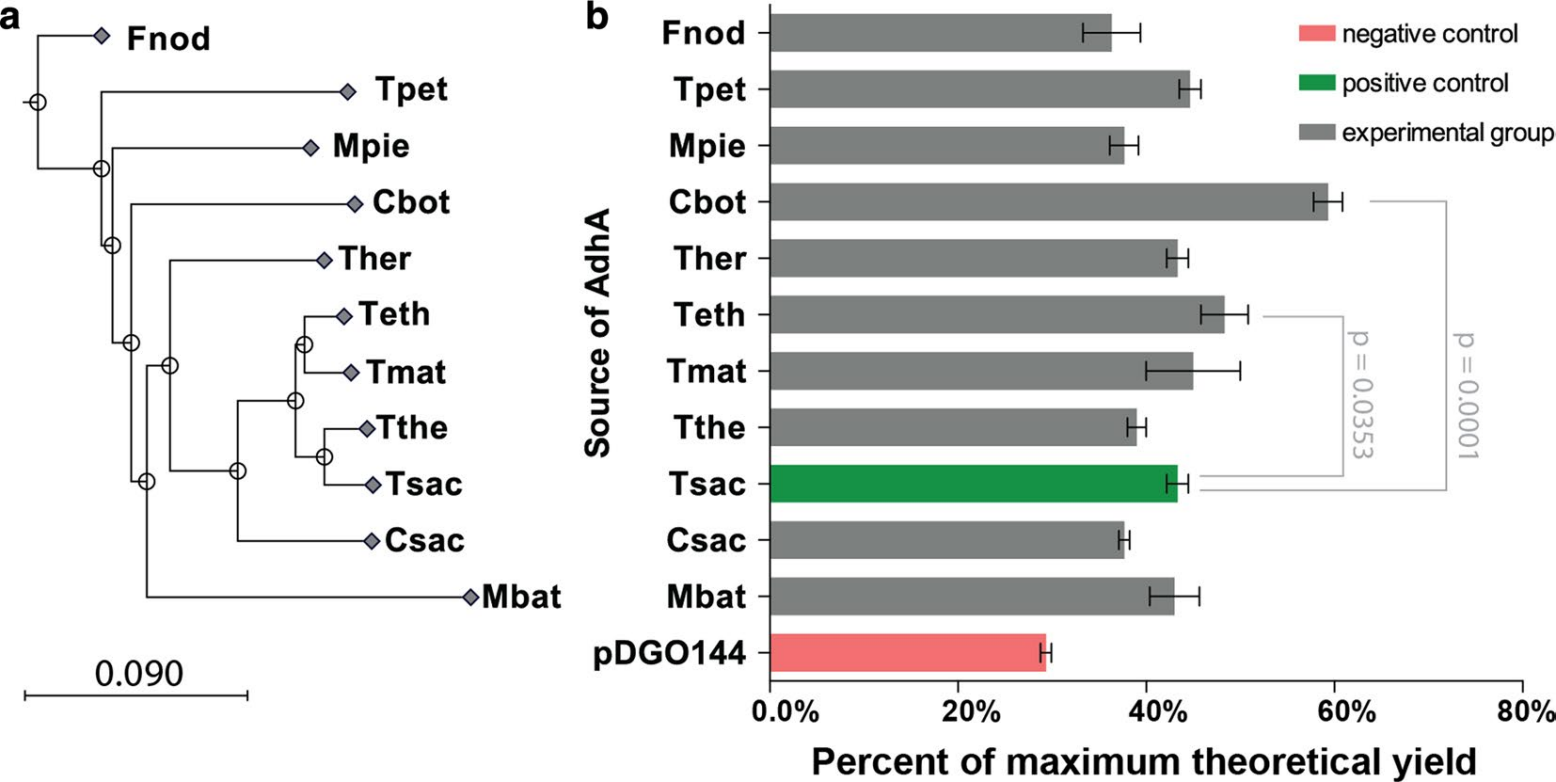
Phylogenetic tree of the AdhA proteins and ethanol yield for 11 *C. thermocellum* strains expressing *adhA* genes from different organisms. **a** The distance-based phylogenetic tree generated from the alignments of the AdhA proteins. **b** Ethanol yield for all of the *adhA*-expressing strains. Strains were cultured in MTC-5 medium containing 4.7 g/l cellobiose and 6 μg/ml thiamphenicol at 55 °C for 72 h. The maximum theoretical yield is 4 mol of ethanol per mole of cellobiose consumed. Data were collected from triplicate experiments. Error bars represent one standard deviation. Wild-type *C. thermocellum* expressing the empty vector pDGO144 (LL1535) was used as negative control (indicated in red), and the strain expressing *T. saccharolyticum adhA* was used as positive control (indicated in green). All of the other 10 strains were shown as the experimental group (indicated in gray). Ethanol yields of experimental group strains were compared to the positive control using a two-tailed unpaired *t* test, and *p* values are reported where significant

Sequences with homology to the *T. saccharolyticum* AdhA were searched using the BLAST (Basic Local Alignment Search Tool) algorithm [13]. AdhA sequences from different organisms were chosen based on protein sequence identity to *T. saccharolyticum* AdhA, with an identity range of 57–90% (Table 1). Most of the selected organisms were thermophilic bacteria with an optimal growth temperature greater than 50 °C as presented in Table 1. *Clostridium botulinum*, a mesophilic bacterium that grows at 37 °C, was also chosen with the intention of exploring the heat stability of AdhA. A phylogenetic tree of AdhA proteins used in this study is presented in Fig. 1.

### Fermentation behavior of *C. thermocellum* strains expressing different *adhA* genes

The 11 *adhA* genes described above, including *T. saccharolyticum adhA*, were cloned into expression plasmid pDGO144 and expressed in wild-type *C. thermocellum*. Fermentation results for all of the strains are presented in Table 2. Wild-type *C. thermocellum* harboring the empty pDGO144 plasmid was used as a negative control strain. Ethanol yield was calculated based on the amount of ethanol produced from the amount of cellobiose consumed. Two-tailed unpaired *T* tests were performed on the ethanol yields of the strains with three biological replicates to assess statistical significance. To evaluate the effect of expressing *adhA* genes in *C. thermocellum,* ethanol yield for each strain was compared to the empty vector negative control. The strain expressing *T. saccharolyticum adhA*, LL1536, had significantly higher ethanol yield than the empty vector control (*p* < 0.0001), agreeing with previous results [2]. The other 10 strains expressing *adhA* genes all had significantly higher ethanol yield compared to the empty-vector control strain (*p* < 0.05). When compared to the positive control that expressed *T. saccharolyticum adhA* (LL1536), two strains exhibited significantly higher ethanol yield: Strain LL1527 expressing *C. botulinum adhA* (*p* = 0.0001) and strain LL1526 expressing *T*. *ethanolicus adhA* (*p* = 0.0353) (Fig. 1). The top two AdhAs in terms of increasing ethanol yield appeared to be evolutionarily distant from each other: *C. botulinum* and *T. ethanolicus*, and we did not observe any correlation between sequence similarity and effect on ethanol production. In general, most of the additional ethanol production came at the expense of acetate production (Table 2). This is consistent with other reports indicating that there appears to be an oversupply of NADPH in *C. thermocellum* [14, 15], and that this can be used to divert C2 flux (i.e., acetyl-CoA) to ethanol in the presence of an NADPH-linked ADH enzyme [8, 9, 16]. Lactate and malate were minor fermentation products. Carbon balances were calculated based on the fermentation end products measured in this study, and they were generally 65–75% closed. The remaining 25–35% of the substrate carbon is likely present in biomass or un-measured fermentation products such as amino acids.

**Table 2:** Fermentation end products of *C. thermocellum* strains expressing different *adhA* genes

Strain ID	Source of *adhA*	Ethanol	Acetate	Formate	Lactate	Malate	Ethanol yield (% maximum theoretical)	Carbon balance (%)
		mM	mM	mM	mM	mM		
LL1527	*Cbot*	32.16 ± 0.57	6.73 ± 0.90	3.51 ± 0.43	0.25 ± 0.04	0.56 ± 0.40	59	71
LL1526	*Teth*	26.27 ± 1.00	9.48 ± 1.64	8.78 ± 1.85	0.33 ± 0.01	0.39 ± 0.12	49	65
LL1525	*Tmat*	24.33 ± 2.50	10.07 ± 1.47	9.59 ± 1.11	0.51 ± 0.21	0.44 ± 0.18	45	63
LL1529	*Tpet*	24.07 ± 0.68	12.81 ± 0.74	4.82 ± 0.91	0.87 ± 0.12	0.50 ± 0.08	45	68
LL1530	*Mbat*	23.23 ± 1.56	12.30 ± 1.56	7.75 ± 1.99	0.94 ± 0.19	0.55 ± 0.10	43	66
LL1536	*Tsac*	23.23 ± 0.62	9.76 ± 0.67	7.53 ± 0.43	0.83 ± 0.00	0.65 ± 0.39	43	61
LL1528	*Ther*	23.21 ± 0.58	10.58 ± 0.43	8.22 ± 0.41	0.71 ± 0.06	0.42 ± 0.01	42	63
LL1532	*Tthe*	21.09 ± 0.37	12.95 ± 1.04	8.43 ± 1.53	0.63 ± 0.06	0.51 ± 0.13	39	63
LL1534	*Csac*	20.29 ± 0.35	12.93 ± 0.64	7.56 ± 0.46	0.74 ± 0.14	0.45 ± 0.03	38	62
LL1531	*Mpie*	20.26 ± 0.60	15.27 ± 0.27	9.08 ± 0.72	0.54 ± 0.06	0.63 ± 0.07	38	65
LL1533	*Fnod*	19.65 ± 1.71	14.21 ± 0.57	7.14 ± 1.88	1.32 ± 0.52	0.85 ± 0.12	36	64
LL1535	NA	15.67 ± 0.22	16.52 ± 0.94	3.04 ± 0.47	3.01 ± 1.17	0.98 ± 0.11	29	64

Data shown here were based on triplicate experiments. Cultures were grown in MTC-5 medium with 4.7 g/l (13.8 mM) initial cellobiose at 55 °C for 72 h. All cultures completely consumed all of the cellobiose initially present in the medium. Thiamphenicol was added at 6 μg/ml for plasmid maintenance. Error is given as one standard deviation, *n* = 3. Rows are ordered by ethanol yield in descending order

## Conclusions

Our results indicate that expressing *adhA* is an effective method of increasing ethanol yield in wild-type *C. thermocellum*, and that this appears to be a general property of *adhA* genes, rather than a property specific to the *adhA* gene from *T. saccharolyticum*. Although most of the *adhAs* studied in this work are from thermophiles, the largest increase in ethanol production came from the *adhA* gene from *C. botulinum*, a mesophile with an optimal growth temperature of 37 °C.
